# Bioinspired Biomaterial Composite for All‐Water‐Based High‐Performance Adhesives

**DOI:** 10.1002/advs.202004786

**Published:** 2021-06-03

**Authors:** Marco Lo Presti, Giorgio Rizzo, Gianluca M. Farinola, Fiorenzo G. Omenetto

**Affiliations:** ^1^ Silklab, Department of Biomedical Engineering Tufts University 200 Boston Avenue, Suite 4875 Medford MA 02155 USA; ^2^ Dipartimento di Chimica Università degli Studi di Bari Aldo Moro via Orabona 4 Bari 70126 Italy; ^3^ Laboratory for Living Devices Tufts University Medford MA 02155 USA; ^4^ Department of Electrical and Computer Engineering Tufts University Medford MA 02155 USA; ^5^ Department of Physics Tufts University Medford MA 02155 USA

**Keywords:** bioinspired biomaterials, mussels, polydopamine, silk, underwater adhesive

## Abstract

The exceptional underwater adhesive properties displayed by aquatic organisms, such as mussels (*Mytilus spp*.) and barnacles (*Cirripedia spp*.) have long inspired new approaches to adhesives with a superior performance both in wet and dry environments. Herein, a bioinspired adhesive composite that combines both adhesion mechanisms of mussels and barnacles through a blend of silk, polydopamine, and Fe^3+^ ions in an entirely organic, nontoxic water‐based formulation is presented. This approach seeks to recapitulate the two distinct mechanisms that underpin the adhesion properties of the *Mytilus* and *Cirripedia*, with the former secreting sticky proteinaceous filaments called byssus while the latter produces a strong proteic cement to ensure anchoring. The composite shows remarkable adhesive properties both in dry and wet conditions, favorably comparing to synthetic commercial glues and other adhesives based on natural polymers, with performance comparable to the best underwater adhesives with the additional advantage of having an entirely biological composition that requires no synthetic procedures or processing.

## Graphical abstract

New mussel‐inspired, high‐performance underwater adhesive is developed using *Bombyx mori* silk fibroin as protein backbone and polydopamine as catechol bearing molecule. This all‐natural synthetic‐step free composition displays in lap‐shearing bonding tests underwater adhesion forces (2.6 MPa) that rival commercial synthetic adhesives.

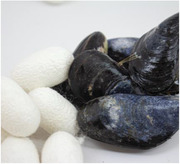

## 1 Introduction

Marine glues and cements are materials with unique chemical and mechanical properties that allow functional performance in challenging environmental conditions such as broad temperature ranges (−20 to +40 °C), chemical variations through fluctuating salinity and moisture, mechanical stressors such as tides, waves, or currents, and the exposure to a whole host of hungry and opportunistic micro‐organisms.

Mussel byssus provides one of the most representative examples of naturally occurring adhesives. Byssus is a polypeptide fiber composed of adhesive proteins,^[^ ^1^ ^]^ specifically conserved oligopeptides^[^ ^2^ ^]^ rich in lysine, hydroxyproline, and dihydroxyphenylalanine (DOPA) residues.^[^ ^3^ ^]^ The adhesive foot of mussels displays a high capacity for crosslinking with its adhesion mechanism involving catechol L‐DOPA residues that are oxidized to reactive quinone moieties, further promoting crosslinkings with other protein‐related residues such as amines and thiols.^[^ ^4^ ^]^ It is widely accepted that oxidation of L‐DOPA residues is required for cohesion, increasing bulk adhesion strength through crosslink formation, while unoxidized DOPA residues are required for adhesion on different surfaces.^[^ ^5^ ^]^ Additionally, mussels have the ability to complement their adhesion capacity by using iron complexes in conjunction with catechols to reinforce the cohesive strength of their ventral byssus.^[^ ^6^ ^]^

Another example of remarkable underwater adhesion mechanism found in nature is exhibited by barnacles, whose anchorage capabilities are displayed on both naturally occurring and man‐made inorganic surfaces.^[^ ^7^ ^]^ Barnacle cement is based on insoluble adhesive nanofibers that consist of numerous protein components that have high *β*‐sheet content.^[^ ^8^ ^]^

The adhesive matrix is composed of small hydrophilic proteins that are responsible for surface‐binding along with larger proteins that have high levels of aliphatic residues, which are thought to comprise the bulk of fibrillar cement.^[^ ^9^ ^]^

This strong and ultraresistant proteinaceous cement^[^ ^10^ ^]^ is rich in amyloid‐like *β*‐sheet domains, which are organized as very compact hydrogen‐bonded structures, aligned perpendicularly to the major polymer axis, and are very stable and able to adhere to any surface.^[^ ^11^ ^]^

More recently, the idea has emerged of leveraging hierarchical self‐assembly of proteins in the design of strong and efficient underwater adhesion materials.^[^ ^9^, ^12^ ^]^

We present here a bioinspired adhesive based on the confluence of the adhesion mechanisms of both mussels and barnacles by investigating a composite constituted of regenerated aqueous silk fibroin (SF) solution and polydopamine (PDA). In order to mimic mussel adhesion, PDA was used as the catechol‐bearing molecule. PDA is a random polymer generated from dopamine oxidative polymerization, with different monomer and oligomer moieties. PDA exhibits a high number of donor and acceptor hydrogen bonding units and aromatic rings,^[^ ^13^ ^]^ creating a branched polymer in which chains strongly interact through hydrogen bonds^[^ ^14^ ^]^ and *π*–*π* stacking.^[^ ^15^ ^]^ Concurrently, to achieve the robust and stable backbones that characterize barnacle cement, *Bombyx mori* SF was chosen as the possible polymer matrix given its ability to assemble in *β*‐sheet domains and because of its ease of processing and functionalization.^[^ ^16^ ^]^ Indeed, SF and barnacle cement are very similar in amino acid composition,^[^ ^12^, ^17^, ^18^ ^]^ and share a common evolutionary origin^[^ ^19^ ^]^ since SF is a fibrous protein–polymer spun by many different types of animals, primarily arthropods, and characterized by extraordinary mechanical properties, such as high tensile strength and extensibility, driven by silk's molecular assembly,^[^ ^20^ ^]^ as well as biological compatibility.^[^ ^1^ ^]^

**Figure 1:**
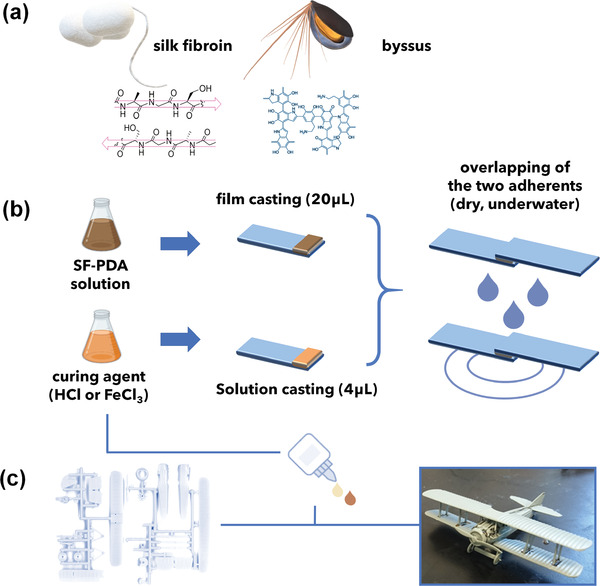
a) Schematic representation of functional components of the blend. Beta‐sheets rich protein backbone from *B. mori* silk fibroin (left) and catechol rich polymer from polydopamine (right) to mimic mussels adhesion. b)Preparation procedure of specimens for lap shearing tests. c) Assembled aircraft model using SF–PDA adhesive with Fe^3+^ curing process as a proof‐of‐concept of SF–PDA glue.

The bioinspired adhesive here presented is designed to mimic mussel adhesive and the cement produced by *Cirripedia* crustaceans by mixing SF and PDA in different proportions through direct dopamine oxidative polymerization in SF aqueous solution. The combination of silk fibroin and polydopamine (SF–PDA) brings together the best adhesive properties of both systems. Specifically, crosslinkable and iron‐chelating DOPA moieties from mussels are introduced by PDA catechols while structural stability is provided by *β*‐sheet rich amyloid‐like barnacle cement via the polypeptide SF backbone (**Figure** **1**a). Further, the adhesion strength is significantly enhanced by FeCl_3_/HCl curing, exploiting both the complexation of catechol units (similarly to what has been observed in mussel adhesives) and the SF aggregation that is observed in acidic environments.^[^ ^21^ ^]^ The formulation of underwater, bioinspired adhesive materials remains a significant problem^[^ ^1^, ^22^ ^]^ given the challenge of developing adhesives that are simultaneously nontoxic, form strong bonds, and are able to set in wet environments, as found in nature.^[^ ^23^ ^]^ The SF–PDA composite adhesive formulation exhibits adhesion properties in dry conditions (up to 2.5 MPa), which are remarkably maintained in underwater environments (up to 2.4 MPa) and favorably compares to all‐natural glues and to the majority of synthetic commercial brands in underwater conditions.^[^ ^22^ ^]^ A demonstrator application of the water‐based SF–PDA glue was carried out by assembling an aircraft model using SF–PDA 200 × 10^−3^ m cured with FeCl_3_ (Figure 1c).

## 2 Results and Discussion

The regenerated aqueous silk fibroin solution was prepared as previously described.^[^ ^18^ ^]^

Different amounts of dopamine (to concentrations of 2 × 10^−3^, 20 × 10^−3^, or 200 × 10^−3^ m) were added to SF solutions (7.3%, i.e., 73 mg mL^−1^) and were left at room temperature for 2 days to allow spontaneous polymerization of dopamine.

To test the adhesion strength of the composite, 20 µL of the resulting SF–PDA solutions were cast on an area of 25 × 10 mm of glass microscope slides (Fisherbrand, Plain, Microscope slides) (Figure 1b).

**Figure 2:**
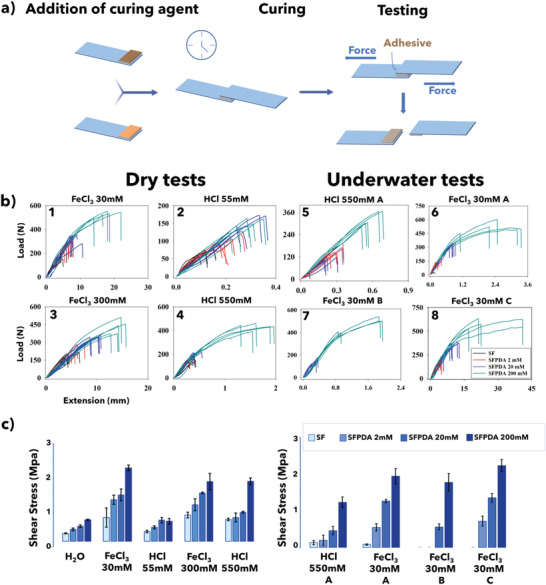
a) Schematic representation of the lap‐shear test. Polymeric adhesives are applied between two adherents and pulled to cohesive failure. b) Load versus extension plots for b1–b4) SF–PDA blends cured with either HCl or FeCl_3_ in dry conditions. b5–b8) Load versus extension plot for SF–PDA blends employing b5,b6) underwater curing, b7) underwater curing and lap shearing, and b8) underwater curing and lap shearing in basic environment. All blends were measured five times for each condition. c) Summary of lap‐shear test results for neat SF and SF–PDA polymer blends after water, acidic, and acidic Fe^3+^ curing processes at different concentrations of curing agents in dry condition (left panel) and underwater conditions (right panel).

After drying (1 h), the films were exposed to different curing agents, namely, 4 µL of i) bidistilled water, ii) HCl (55 × 10^−3^, 550 × 10^−3^ m), or iii) FeCl_3_ (30 × 10^−3^, 300 × 10^−3^ m) and a second microscope slide was then placed atop the first one to create a lap shear joint. A binder clip was used to hold together the adherents for an overnight curing period. Subsequently, the two microscope slides were used to perform lap shear tensile strength tests,^[^ ^23^, ^24^ ^]^ where the two adherents are controllably pulled apart by a material testing system, thus enabling the quantification of bonding strength. In all the experiments performed, the bond failure was found to be of a cohesive nature since the polymer was distributed on both substrates after bond failure (**Figure** **2**a). Each measure was repeated five times.

HCl was used as a curing agent to prevent the oxidation of catechols to quinones, which is the dominant condition at pH above 5.5^[^ ^25^ ^]^ and also to act as a reference for the sample treated with FeCl_3_, which is also acidic and has the same pH. FeCl_3_ is essential when triggering catechols’ chelating ability toward Fe^3+^ that subsequently leads to strong complexes and, thus, high adhesion strengths while creating an acidic environment at the same time, similarly to the HCl case.^[^ ^26^ ^]^ Additionally, FeCl_3_ favors SF aggregation via the coordination of polar amino acids such as tyrosine and serine residues.^[^ ^27^ ^]^

Figure 2b summarizes the tensile strength testing results as a function of the different curing agents used. Neat PDA fails to stick the glass slides together without SF. In fact, PDA essentially acts as an efficient crosslinker between the reactive moieties of SF but does not result in any appreciable cohesion phenomena.

Neat SF displays poor adhesion properties, comparable to most all‐natural glues reported in the literature,^[^ ^28^ ^]^ with 20 µL of 7.3% SF solution showing an adhesion strength of about 0.2 MPa.

An increase of adhesion performance up to 1 MPa was previously reported when the tyrosines in silk are converted into DOPA moieties.^[^ ^29^ ^]^ Moreover, other authors have proposed the combination of silk and polydopamine by coating silk fibers in order to create an adhesive cement^[^ ^30^ ^]^ or materials for wound dressing.^[^ ^31^ ^]^

Recent reports ^[^ ^32^, ^33^ ^]^ of dopamine bound to SF in solution to the generate a hydrogel for wound healing applications offer an evaluation of the biocompatibility of the blend composition. The addition of dopamine, which freely self‐polymerizes in SF solution (Figure S1, Supporting Information), increases the adhesion properties of casted SF films as the dopamine concentration rises. The maximum concentration used in the experiments (200 × 10^−3^ m) increased the adhesion strength of pure SF threefold reaching a value of 0.6 MPa (Figure 2c left panel).

The addition of HCl further improved the adhesive strength of the samples (Figure 2b2,b4). In particular, SF–PDA 200 × 10^−3^ m cured with the highest HCl concentration (HCl 550 × 10^−3^ m) showed adhesion strength of up to 2 MPa (Figure 2c left panel), exceeding the strength of most reported natural adhesives.^[^ ^34^ ^]^

Adhesion strengths of over 1 MPa (≈145 pounds per square inch (psi)) are often considered to fall in the so‐called “high‐strength bonding range,”^[^ ^35^ ^]^ thereby enabling applications in several fields.^[^ ^24^ ^]^ By comparison, synthetic mussel proteins produced by genetically modified bacteria achieve appreciable adhesion strengths measuring up to ≈1 MPa.^[^ ^36^ ^]^

High adhesion strengths can also be attained using Fe^3+^ as the curing agent as shown in (Figure 2b1,b3), reaching 2.0 MPa with the use of FeCl_3_ at 30 × 10^−3^ m concentration.

It has been previously established that SF solutions rapidly gelify when exposed to acidic environments^[^ ^37^ ^]^ due to the neutralization of charged residues at low pH values causing proximal chains to approach each other and thus causing their entanglement through hydrophobic interactions.^[^ ^38^ ^]^ A similar increase in adhesion strengths was also observed in SF–PDA systems (Figure 2c left panel), with additional effects due to enhanced PDA surface interaction through hydrogen bonds arising from the protonation of catechol moieties.^[^ ^39^, ^40^ ^]^

Proton bridging is a ubiquitous interaction system between two atoms competing for the same proton, thus resulting in a tightly bound binary complex. Such interaction is crucial in almost all proteins and other biological molecules.^[^ ^41^ ^]^ Thus, the presence of PDA in the acidic environment could lead to µ–H^+^ bridging between quinones and catechols enhancing crosslinking and thus favorably affecting the adhesive strength of the composite as supported by viscosity measurements (see Table S1 in the Supporting Information).

The choice of FeCl_3_ as a curing agent is inspired by the adhesion mechanism exhibited by mussels that accumulate Fe^3+^ and use it as a crosslinker between catechol units. Iron coordination requires deprotonated catechol units and thus chelation depends on pH values. In fact, only catecholate units can efficiently bind metals, whereas their protonated form interacts with other polar moieties through hydrogen bonding. This is supported by experimental evidence demonstrating that both the SF–PDA 200 × 10^−3^ m cured with 550 × 10^−3^ m HCl and with 300 × 10^−3^ m FeCl_3_, which have nearly the same pH (0.96), exhibit adhesive strengths that are almost the same: specifically, 1.2 and 1.3 MPa, respectively (Figure 2c left panel). On the contrary, in SF–PDA 200 × 10^−3^ m samples cured with FeCl_3_ 30 × 10^−3^ m (pH 1.96), the adhesion strength exceeds 2 MPa probably due to the higher ratio Fe^3+^/H^+^ since both compete for interaction with catechol. Moreover, the higher ratio catechol/Fe^3+^ favors the formation of tris‐complexes, which effectively enhances the crosslinking.

As an additional proof of the wide applicability of FeCl_3_ cured SF–PDA blends, we have also performed additional lap‐shear tests on different substrates, observing maximum lap shear values 4.7, 2.2, and 0.8 MPa on steel, aluminum, and plywood, respectively (see Figure S2 in the Supporting Information).

**Figure 3:**
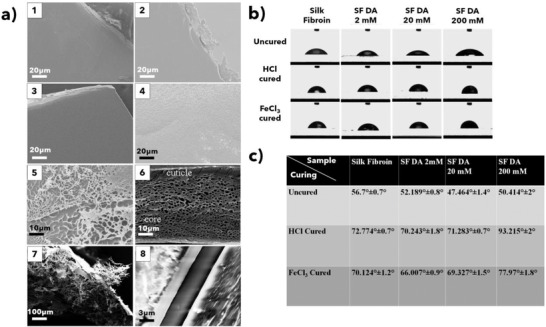
a1–a4) SEM images and relative film insets of different SF–PDA blends. a1) SF film; a2) SF–PDA 2 × 10^−3^ m; a3) SF–PDA 20 × 10^−3^ m; a4) SF–PDA 200 × 10^−3^ m; a5) alveolar porous structure of SF–PDA 200 × 10^−3^ m after curing with FeCl_3_ 30 × 10^−3^ m and lap‐shear test; a6) natural byssal plaque of *Mytilus edulis*; a7) alveolar structure of SF–PDA 200 × 10^−3^ m cured with FeCl_3_ 30 × 10^−3^ m (not subjected to lap‐shear test) with a lower magnification; a8) cross‐sectional SEM of SF–PDA 200 × 10^−3^ m between two glass slides. Markers are reported for each image. a6) Reproduced with permission.^[^ ^1^ ^]^ Copyright 2005, Taylor & Francis. b,c) Static contact angles of SF and SF–PDA films uncured or cured either with 4 µL of HCl 55 × 10^−3^ m or FeCl_3_ 30 × 10^−3^ m against bidistilled water.

The morphology of these blends was also characterized by scanning electron microscopy (SEM) analysis revealing that, after the iron curing process, the resulting matrix resembles natural byssus (**Figure** **3**a6). SEM images of dried SF–PDA adhesives before (Figure 3a1–a4) and after curing (FeCl_3_) and detachment of the adherends (Figure 3a5) are also reported. Before the curing process, the films display increasing roughness with an increase of dopamine percentage. A comparison between the SF–PDA glue and mussel plaque^[^ ^1^ ^]^ after curing and detachment in (Figure 3a5) illustrates the similarities in morphology and pore dimension. Porous structures are also visible in SF–PDA cured films not subjected to lap‐shear test thus revealing that it originates from the composition and curing procedure (Figure 3a7). This solid foam‐like internal structure is reminiscent of the strategy adopted by mussels (Figure 3a6) and other marine organisms to increase the elasticity of the adhesive and minimizing the abruptness of the elastic modulus mismatch between the rigid particles and the flexible cement.^[^ ^42^ ^]^ The cross‐section of the two glass adherends glued together shows that the thickness of the adhesive layer is less than 3 µm (Figure 3a8).

The underwater adhesion properties^[^ ^1^ ^]^ of this blend were then investigated by using the protocol described by Payne and co‐workers for chitosan–PDA blends.^[^ ^5^ ^]^ Experiments were performed for SF–PDA blends cured with H_2_O, HCl 550 × 10^−3^ m, or FeCl_3_ 30 × 10^−3^ m as described previously. Immediately after the addition of curing agent, the overlapped surfaces were clipped together with binder clips and kept immersed in bidistilled water for 24 h.

After 24 h of submersion, all three samples series were measured in different conditions either extracting them from the solution and performing the lap shearing test in dry conditions, labeled as “A,” or carrying out lap‐shear tests underwater labeled as “B” (Figure 2b5–b8). An underwater adhesion experiment was also performed with basic water (pH 9) to determine the effects of pH on adhesion (labeled as C).

For the measurements, “B” (Figure 2b7) and “C” (Figure 2b8) only one concentration of FeCl_3_ (30 × 10^−3^ m) was used as the curing agent.

In underwater environments, water cured SF–PDA samples spontaneously detached after 24 h of water immersion. Neat SF also failed to attach the adherents underwater even after curing with HCl or FeCl_3_. Only SF–PDA blends were able to resist in underwater environments.

Measurements showed (Figure 2c right panel) that acid curing by HCl is required to obtain underwater adhesion, which improves by increasing dopamine concentration up to 1.5 MPa in SF–PDA 200 × 10^−3^ m.

Better results can be obtained with FeCl_3_ curing (Figure 2c right panel), where underwater adhesion strengths as high as 2 MPa can be observed. When lap shearing measurements are performed underwater, the samples with the highest concentration of PDA (200 × 10^−3^ m) showed adherent strength (1.9 MPa) that were almost entirely unaffected by the different measurement conditions, while the other samples with lower PDA concentrations showed dramatically reduced or negligible adhesion.

The best results were obtained in basic underwater conditions (“C”), where the SF–PDA (200 × 10^−3^ m) exhibits adhesion strengths of up to 2.4 MPa, the highest strengths recorded in these experiments. It is well known in adhesive technology that both adhesive and cohesive interactions are required to join two substrates.^[^ ^13^ ^]^ In our system, the leading moiety involved is the catechol ring responsible for surface adhesive contacts but which, once oxidized to quinone, undergoes oxidative crosslinking reactions generating cohesive bonds within the matrix as depicted in Scheme S1 in the Supporting Information.

The reduction in adhesive strength of HCl (550 × 10^−3^ m) cured underwater when compared to dry samples may be attributed to the dilution of HCl upon immersion of the adherents in water, which, as stated before, is likely to cause loss of adhesion via hydrogen bonds.^[^ ^43^ ^]^

Samples cured with FeCl_3_ showed greatly improved underwater performance when compared to HCl curing.

The improvement in adhesion strength of FeCl_3_ cured samples against HCl cured samples can be attributed to the manifold action of FeCl_3_. Fe^3+^ interaction with SF–PDA which occurs via chelation, redox chemistry or, more realistically, a combination of both. Notably, only after FeCl_3_ curing both the 5,6‐dihydroxyindole and indole‐5,6‐quinone forms are found in SF–PDA films, while is not the case for HCl curing (Figures S3 and S4, Supporting Information). Moreover, a protocol based on first curing and subsequently pouring the adherent into water could be held responsible for promoting adhesion first, favored by low pH, and only subsequently, after dilution, the oxidation/coordination that triggers the chelating ability for iron with the formation of mono‐ to bis‐ and/or tris‐catecholate complexes,^[^ ^44^, ^45^ ^]^ which leads to an increase in cohesion.^[^ ^46^, ^47^ ^]^ In other words, catecholic molecules first adhere to a substrate, while oxidative/coordination‐mediated curing, which improves cohesion of the matrix, only takes place afterward. This mechanism is reminiscent of the natural mussel foot process whereby mussels acidify the environment around their foot to pH = 2 to ensure that reducing conditions are present during the adhesive deposition.^[^ ^48^ ^]^ However, after glue stabilization and foot detachment, mussels induce rapid changes in this environment raising the pH to 8, thus ensuring efficient adhesions due to catechol deprotonation and hence strong Fe^3+^ chelation.

The adhesive results reported in the basic underwater conditions in these experimental trials provide better values than those measured in dry conditions and, more generally, are among the highest values reported for an underwater adhesive. This is particularly compelling given that this glue is entirely based on natural materials and SF modification with dopamine is achieved through a spontaneous polymerization in an aqueous environment not requiring further purification procedures. The increased adhesive properties in basic conditions can arguably be attributed to a combination of deprotonation of catechol moieties leading to a higher degree of coordination with iron and to increased quinone formation and oxidative crosslinking.^[^ ^13^ ^]^

The curing process also causes an increase in hydrophobicity of SF–PDA blends that were assessed by contact angle measurements (Figure 3b) where a PDA coated glass slide showed total wettability since the drops were found to completely spread on the surface (data not shown). Accordingly, as the PDA content increased so did the wettability of the SF–PDA blends with a dopamine content of 2 × 10^−3^ and 20 × 10^−3^ m. The 200 × 10^−3^ m displayed a turnover with a slight decrease of wettability, but still superior to the unmodified SF (Figure 3b).

After acidic curing of the film with HCl, the contact angles in each mixture increased from 14° to 43° showing an increase in the surface hydrophobicity. The highest modification was observed in SF–PDA (200 × 10^−3^ m) whose contact angle reached 93.22°. Similar results are obtained when using FeCl_3_ as the curing agent where the increase in the acid environment caused by Fe^3+^ ions causes corresponding increases in contact angles as for HCl. Slightly increased wettability compared to HCl curing was attributed to the presence of a significant amount of Fe^3+^ ions in the SF–PDA blend (Figure 3c).

The contact angle results reveal that the surface wettability of cast SF films is affected by PDA content^[^ ^31^ ^]^ and, more markedly, by the curing procedure of the casted film.

The increase in hydrophobicity of a peptidic polymer is primarily related with exposed functional groups, arising from the structural reorganizations of macromolecules.^[^ ^49^, ^50^, ^51^ ^]^

The fact that a hydrophilic protein produces a hydrophobic film after exposure to an acidic environment suggests that SF macromolecules adopt a conformation that maximizes the exposure of hydrophobic groups and minimizes the surface content polar groups, decreasing the free energy at the polymer–substrate interfaces.^[^ ^52^ ^]^

Remarkably, this behavior mimics the natural phenomenon whereby the high proportion of hydrophobic residues found in mussels (especially Mfp‐3/5, the richest DOPA‐containing mussel proteins) creates a microenvironment that shields DOPA from oxidation and leads to hydrophobic interactions that appear to enhance DOPA wet‐adhesion on substrates.^[^ ^53^ ^]^

**Figure 4:**
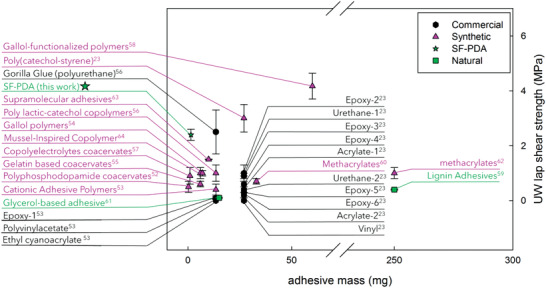
Plot reporting failure (MPa) against mass (mg) of recently reported adhesives in underwater conditions. The reported values refer to commercial (black exagon), synthetic (pink triangles), or natural‐based (green squares) adhesives.

Measurements of water contact angle values on protein surfaces have been reported to be correlated to their adhesion properties. Specifically, a water contact angle (*θ*_W_) of 65° has been shown to be the boundary condition separating adherent (*θ*_W_ > 65°) versus nonadherent (*θ*_W_ < 65°) protein materials.^[^ ^49^, ^54^ ^]^ To further contextualize the performance of the SF–PDA blend glue, the results are compared with other adhesion values reported in the literature^[^ ^55^, ^56^, ^57^, ^58^, ^59^, ^60^, ^61^, ^62^, ^63^, ^64^, ^65^, ^66^, ^67^, ^68^ ^]^ (**Figure** **4**). Among all commercial underwater adhesives, the best compromise between dry and underwater performance is polyurethane‐based glues (i.e., Gorilla Glue^[^ ^68^ ^]^), with reported strengths of 2.8 MPa in dry conditions and 2.5 MPa in underwater conditions (when 13.5 mg of adhesive is applied). Among synthetic adhesives, North et al. have proposed a system that reaches 3 MPa underwater albeit the adhesive mixture is applied using chloroform as the solvent.^[^ ^22^ ^]^

Although these examples perform efficiently as adhesives, they are synthetic and require application procedures that involve the use of harmful reactants and solvents. Moreover, the amount of adhesive applied for the test was up to 13 times higher than SF–PDA blends presented herein, which employ only 1–2 mg of adhesive. More specifically the amount used in the experiments here presented was from 4^[^ ^69^ ^]^ to 100^[^ ^70^ ^]^ times lower than other reported adhesives, making this system quite simple from the standpoint of synthesis, application, and curing conditions.^[^ ^67^ ^]^

For naturally based adhesives, which are more sparsely discussed in the literature the highest strengths displayed for lap shearing tests are 0.4 MPa for dry conditions^[^ ^28^ ^]^ (employing 14 mg of neat SF as the adhesive) and 0.4 MPa for underwater conditions^[^ ^5^ ^]^ (employing 100 mg of a chitosan–PDA blend) further validating the appealing properties of this SF–PDA blend.

## 3 Conclusions

In conclusion, the inspiration provided by the adhesion and anchoring mechanisms of marine organisms has driven the preparation of an all‐natural, water‐based adhesive blend using SF and dopamine, which displays outstanding properties in dry and underwater conditions. The preparation and application of the blend employ benign chemistries that require no synthetic steps nor any use of organic solvents from the extraction process from raw materials all the way to adhesive application and has properties that rival or exceed natural and synthetic systems with the underwater adhesion strength exceeding, in certain cases, adhesion performance rates in dry conditions all while using small (1–2 mg) quantities of adhesive.

The composite also provides insights for the adhesive strategies adopted by marine organisms, presenting a simple but effective reduction to practice that recapitulates many of the features found in natural adhesives like metal chelation, catechol interaction, and pH‐induced hydrophobic enhancement.

## 4 Experimental Section

### Materials

Dopamine hydrochloride (Sigma‐Aldrich, >99.9%), anhydrous ferric chloride FeCl_3_ (Sigma‐Aldrich, ≥99.99% trace metal basis), hydrochloric acid, HCl (Sigma‐Aldrich, reagent grade, ≥37%), and sodium carbonate (Na_2_CO_3_) were used without any further purification. Raw *B. mori* silk cocoons were purchased from Tagima Shogi (Japan).

### Silk Fibroin Solution Preparation

Silk fibroin solution was prepared as previously reported.^[^ ^15^ ^]^ In brief, *B. mori* silk cocoons were cut and boiled for 30 min in Na_2_CO_3_ (0.02 m) to remove sericin. LiBr solution (9.3 m) was added to the overnight‐dried silk fibroin and stored at 60 °C to dissolve fibers into an aqueous solution. Pure silk solution (≈8%) was collected after dialysis (Fisherbrand, MWCO 3.5 K) for 3 days. A proper dilution was used to obtain the 7.3% SF solution used for adhesion experiments.

### Silk–Polydopamine Blend Preparation

Silk fibroin solution (7.3%) was modified with the addition of dopamine hydrochloride at a final concentration of 2 × 10^−3^, 20 × 10^−3^ , and 200 × 10^−3^ m. The solution was stored in the fridge for 2 days allowing the spontaneous polymerization of polydopamine detectable by a browning of the solution.

### Mechanical Tests

Adhesion tests were performed on an Instron 3366 testing frame equipped with a 1000 N load cell (Norwood, MA, USA) following a modified version of ASTM method.^[^ ^1^ ^]^ Adherents mounted in the tension grips were subjected to single lap‐shear testing, repeating each measure at least five times. 1000 N load cell equipped Instron was programmed to move the grips apart at 0.5 mm s^−1^ rate. Tests were stopped when adhesive bond ruptured and the maximum shear strength peak was divided by the bound area to give adhesion. Glass (Fisherbrand, Plain, Microscope slides, 25 × 75 × 1.0 mm) samples were used as test surface materials for adhesion, following the ASTM standard method to properly cut materials.

### SEM Analysis

SEM was performed with a LEO Gemini 982 Field Emission Gun (Thornwood, NY, USA). An operating 5 kV voltage for morphology analysis was applied. Surfaces of specimens were coated with gold sputtering for 45 s at 15 mA in Argon atmosphere. Films (500 µL) were prepared pouring of different solutions in Teflon plates and allowed to air‐dry.

### Contact Angle Analysis

Static contact angles of SF, SF–PDA, and PDA films cast on Fisherbrand, Plain, Microscope slides, 25 × 75 × 1.0 mm against bidistilled water were measured by the sessile drop method using a ramé‐hart instrument co., PO Box 400, Netcong, NJ, USA, at 22 °C and about 65% relative humidity. The wettability of neat PDA layers was assessed on samples prepared by placing a glass slide in a phosphate buffer (pH 8.5) with dopamine hydrochloride (5 × 10^−3^ m) for 2 days to allow the formation of a thin layer of PDA onto the glass slide by dopamine polymerization. To assess the wettability of SF–PDA blends, films were prepared as for the lap shear measurement and tested before and after the curing procedures with 4 µL of either HCl (55 × 10^−3^ m) or FeCl_3_ (30 × 10^−3^ m). These data were determined after a reasonable equilibration time (1 min). H_2_O drops (7 µL) were applied with a microliter syringe. All reported contact angle values were the average of at least five measurements taken at different locations on the film surface.

### Statistical Analysis

The experimental data of mechanical lap‐shear tests were presented as mean values ± standard deviation (SD) of 5 (*n*) samples for each blend and condition and were statistically analyzed to determine the significance of differences determined by paired *t*‐tests with a two‐tailed distribution and one‐way analysis of variance (ANOVA) using Microsoft Excel. Differences were considered to be statically significant at *p* < 0.05.

## Conflict of Interest

The authors declare no conflict of interest.

## Supporting information

Supporting Information

## Data Availability

The data that support the findings of this study are available from the corresponding author upon reasonable request.
