# Supplementary material for: Bioinspired Biomaterial Composite for All‐Water‐Based High‐Performance Adhesives
Source: Adv Sci (Weinh). 2021 Jun 3;8(16):2004786. doi: 10.1002/advs.202004786 (PMC8373158; doi:10.1002/advs.202004786)
Supplement: Supplementary file 1 — Supporting Information [file ADVS-8-2004786-s001.pdf]

© 2021 Wiley-VCH GmbH

ADVANCED SCIENCE
Open Access

Supporting Information

for *Adv. Sci.*, DOI: 10.1002/advs.202004786

Bioinspired Biomaterial Composite for All-Water-based High-Performance Adhesives

Marco Lo Presti, Giorgio Rizzo, Gianluca Maria Farinola, and Fiorenzo G. Omenetto**

Supporting Information

Bioinspired Biomaterial Composite for All-Water-based High-Performance Adhesives

Marco Lo Presti, Giorgio Rizzo, Gianluca Maria Farinola*, Fiorenzo G. Omenetto*

1. UV-vis characterization

Figure S1 shows UV-Vis spectra of DA polymerization after 2 days in presence and in absence of SF
In both cases, solutions were buffered at pH 8.0 and diluted 1:100.
The case of SF-PDA 200 mM is not reported due to the high absorbance of dopamine.

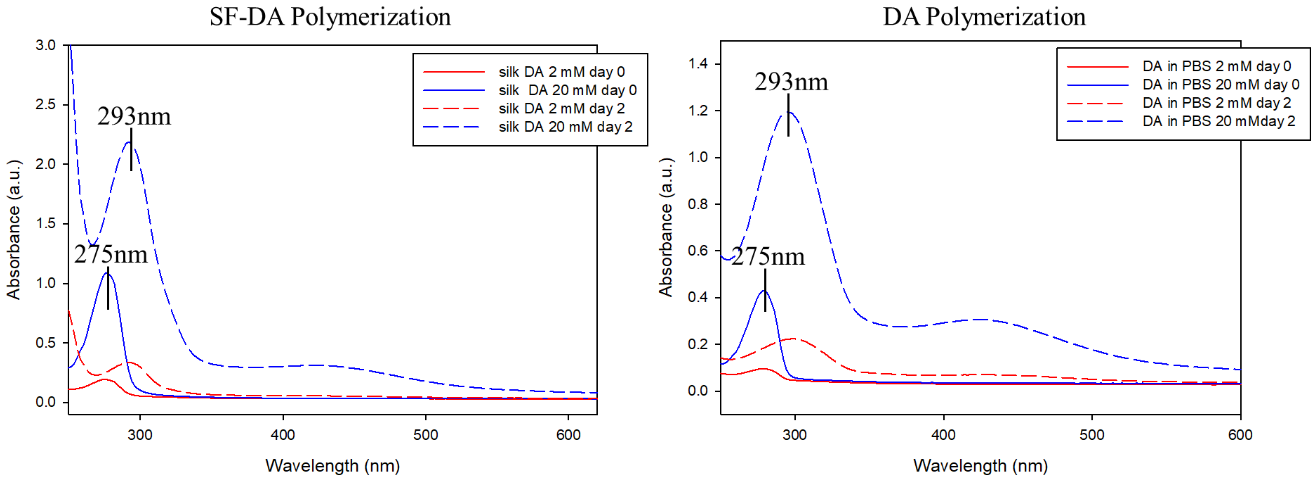

Figure S1. UV-Vis spectra of DA 2 mM (red) and 20 mM (blue) before (continuous line) and after two days polymerization (dashed line). Polymerization was performed in the presence of SF (left) and in PBS (right).

DA polymerization absorbance profile doesn't change in presence of SF. In both cases, there is a shift at 293 nm indicating the formation of 5,6- dihydroxyindole[1] and a broad increase around 450 nm responsible for the formation of dopamine quinone.[2]

2.Viscosity characterization

The addition of DA to SF solution doesn't change the viscosity, but its polymerization causes it to decrease slightly, as already reported in similar systems.[3]
This, supported by the UV-Vis characterization, suggests that, during the polymerization of DA, the formation of PDA is favored over cross-linking with SF. At this stage, this is desirable because, otherwise, the gelation of the solution would occur making the glue ineffective.
The addition of HCl or $FeCl_3$ to the solution causes an increase in viscosity that is higher in presence of PDA, revealing that the curing procedure is responsible for activation of the cross-linking between silk and PDA. It is important to underline that although the viscosity results are in accordance with this hypothesis, they were obtained with a procedure that is not perfectly comparable to the adhesive curing, since they are performed in solution while the

curing of the adhesive occurs on a dried SF film thus involving higher concentrations of SF, PDA and curing agent.

Table S1. (Viscosity of SF and SF-PDA blends recorded over three days and after the addition of either HCl or $FeCl_3$)

	Day 0	Day 1	Day 2	Day 2 + HCl	Day 2 + Fe^{3+}
SF	11.4 ± 0.4 cP	11.5 ± 0.3 cP	11.4 ± 0.3 cP	11.8 ± 0.3 cP	12.5 ± 0.9 cP
SFDA2	11.9 ± 0.9 cP	11.4 ± 1.5 cP	9.1+/-0.5 cP	12. ± 0.7 cP	14.2 ± 1.1 cP
SFDA20	11.58 ± 1 cP	11.4 ± 0.45 cP	10.4 ± 0.35 cP	12.5 ± 0.65 cP	14.6 ± 0.5 cP
SFDA200	11.7 ± 1.6 cP	11.7 ± 0.35 cP	11.1 ± 1.93 cP	13.8 ± 0.55 cP	14.9 ± 1.1 cP

3. Lap-shear tests on different materials

Since the curing with $FeCl_3$ 30 mM has given the best results, additional lap-shear tests were performed in dry conditions on different materials using this type of curing.

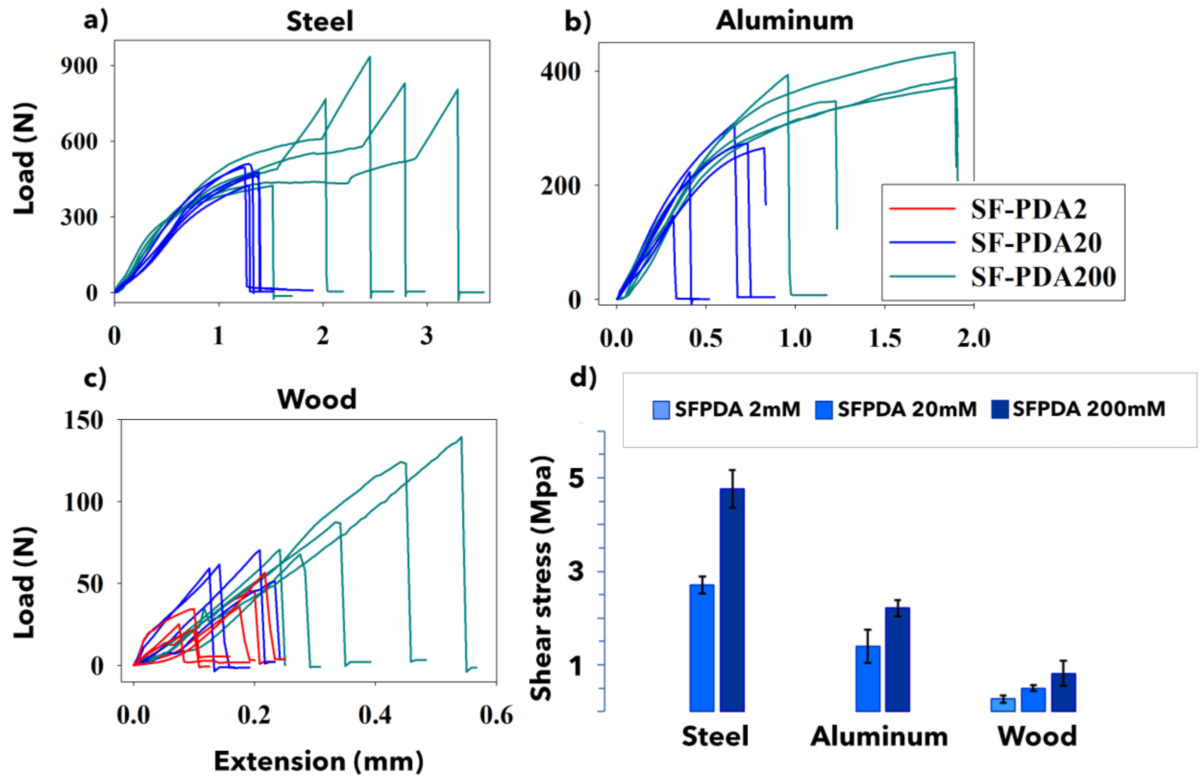

Figure S2. Load vs extension plot of SF-PDA blends on a) steel, b) aluminum and c) plywood specimens. d) histogram summarizing the shear stress (*MPa*) of SF-PDA blends on the different substrates.

Figure S2 a, b and c show load vs extension plot of SF-PDA blends on stainless steel, aluminum and wood adherent, respectively. SF without PDA wasn't able to stick those kinds of materials since the adherends were detached before the test. SF-PDA 200 displayed a maximum load up to 4.7, 2.2 and 0.8 *MPa* on steel, aluminum and wood respectively. Lower values were recorded for SF-PDA 20 which displayed 2.7, 1.4 and 0.5 *MPa*. Metal samples of SF-PDA 2 blend spontaneously detached before the measure and displayed a load value of 0.27 *MPa* on wood adherends (figure S2d).

4. Proposed cross-linking

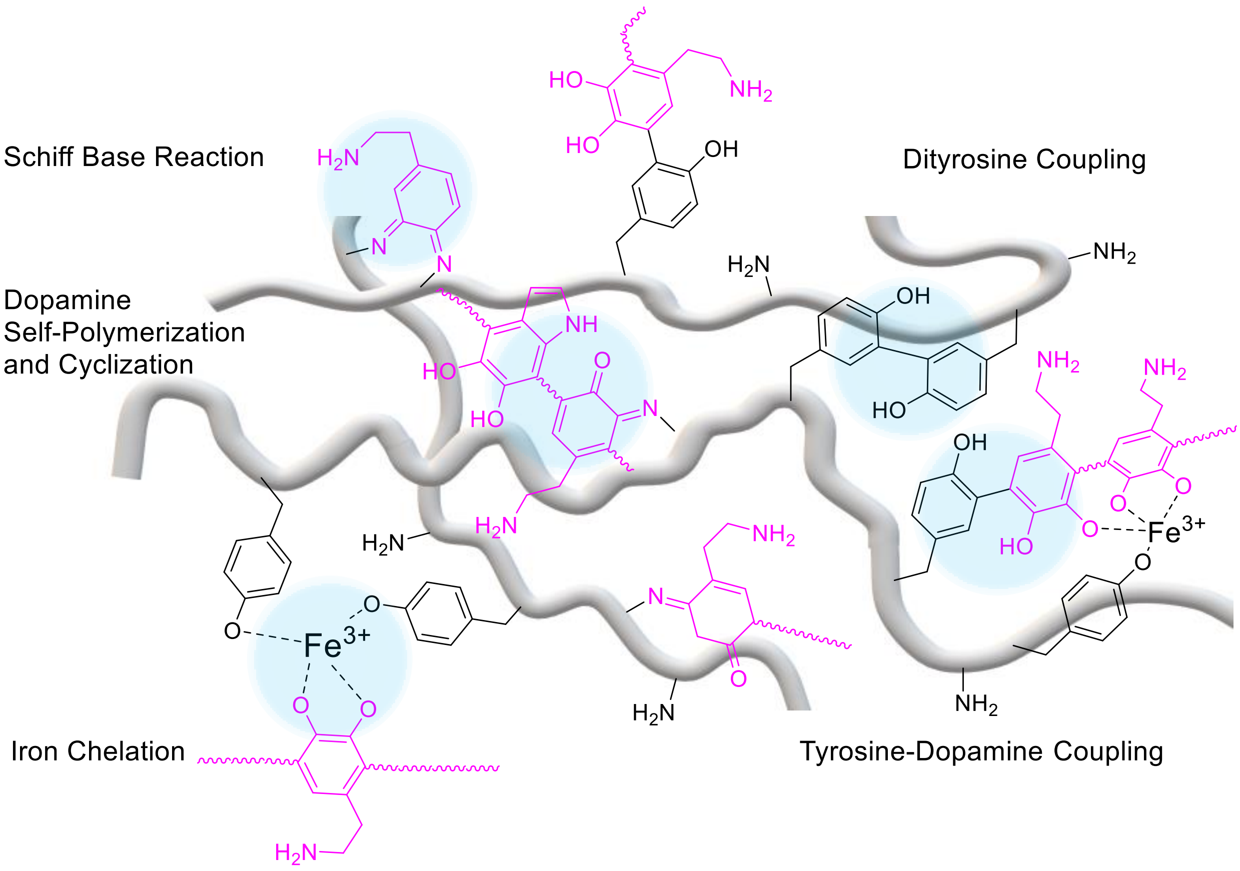

Scheme S1. Proposed interactions between SF moieties (black) and dopamine/PDA residues (pink).

Scheme S1 depicts the possible interactions involved in SF-PDA blends after curing: tyrosine-dopamine and dityrosine coupling as well as Schiff base reactions and iron chelation are highlighted.

5. ATR-FTIR characterization

Figure S3 reports the ATR corrected spectra of SF, SF-PDA 200 after polymerization, SF-PDA 200 after HCl curing, SF-PDA 200 after $FeCl_3$ curing and dopamine. SF shows a broad absorption band centered at 3275 cm^{-1} but is shifted at 3211 cm^{-1} and intensified in SF-PDA blends. The band is the sum of the absorption peaks at 3211 cm^{-1}, 3056 cm^{-1}, 2927 cm^{-1} which corresponds to stretching vibration -NH, -OH belonging to catechol group, and aromatic -CH, respectively.[4]

The shift to lower wavenumbers in SF-PDA blends suggests H-bond interactions between SF and PDA,[4] and its increased intensity indicates a higher amount of Hydrogen bonding donor/acceptor units that translates in higher adhesion capacity.
Other relevant peaks are the amide I band of SF at 1642 cm^{-1} amide II band at 1520 cm^{-1}.[5] The amide II band (N–H bending vibration) is overlapped to the signal at 1502 cm^{-1} of DA attributed to C=C stretch vibration in the benzene ring. Amide II band is stronger than amide I in all cured SF-PDA blends suggesting the presence of aromatic structures, such as indole derived PDA polymerization fragments.
The C–N bending vibration peak appeared near 1236 cm^{-1}, which was established to be the characteristic absorption of SF amide III. This band appears weakened and broadened after HCl curing. The amide III band is usually divided into two bands attributable to SF secondary structure at 1258 cm^{-1} (random coil) and 1229 cm^{-1} (β-sheet)[4] suggesting that HCl treatment causes random coil transitions.

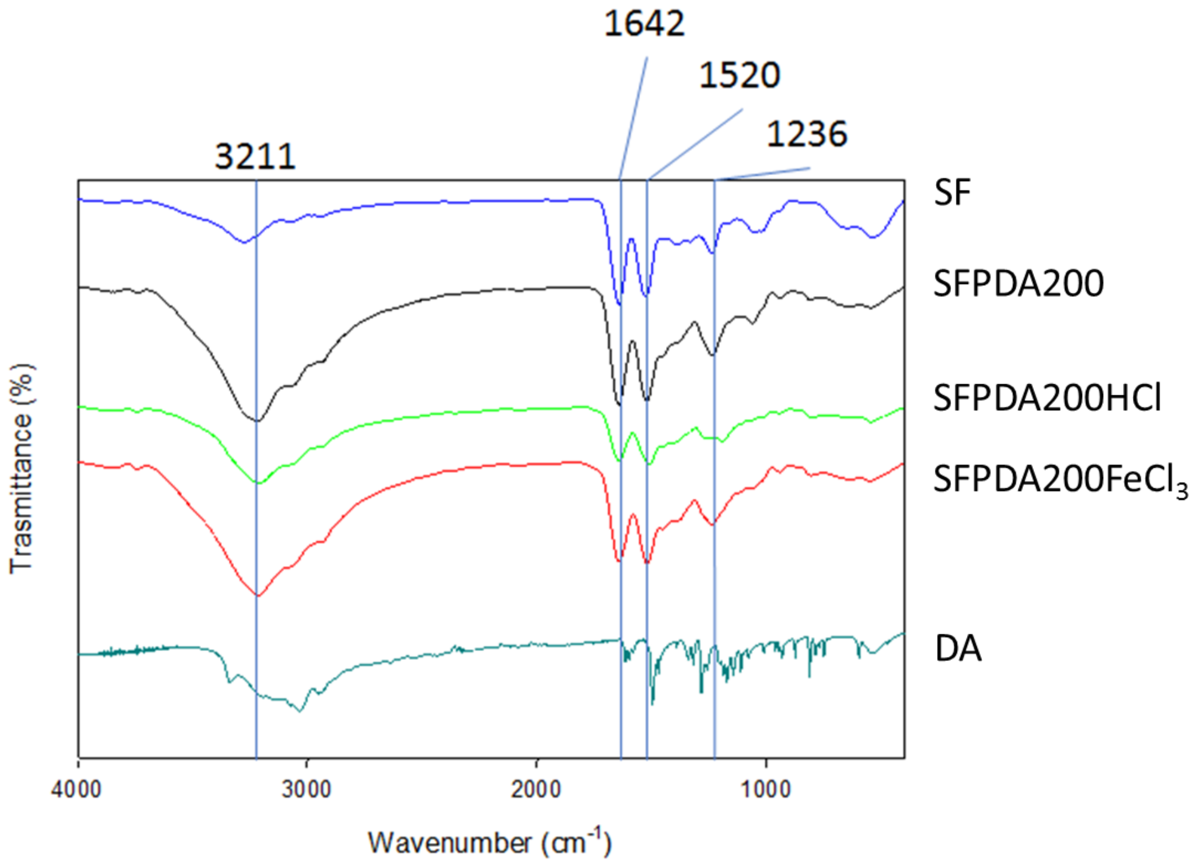

Figure S3. (FTIR-ATR spectra of SF (blue line), SF-PDA 200 (black line), SF-PDA 200 HCl cured (green line), SF-PDA 200 $FeCl_3$ cured (red line) and dopamine molecule (cyan line).)

Figure S4 shows superimposed spectra of SF and SF-PDA to appreciate the modifications induced by different curing procedures. A peak at 1370 cm^{-1} is found in SF-PDA 200 and SF-PDA 200 Fe^{3+} and is responsible for indole ring stretching mode.[6] Moreover, the peak at 1453 cm^{-1} in SF-PDA and SF-PDA Fe^{3+} correspond to the specific structure of polyindole.[7],[8]
Both these peaks disappear after HCl curing. That is attributed to the structural transition of 5,6-dihydroxyindole to indole-5,6-quinone (which is known to be induced by HCl treatment), which usually translates in the appearance of a new band at 1740 cm^{-1} corresponding to ketone and the weakening or disappearance of peaks at 1453 cm^{-1} and 1110 cm^{-1}.[9]

Notably, in our system we found the disappearance of peaks at 1453 and 1110 cm^{-1} in HCl cured blend but without the appearance of the band at 1740. Instead, we found the band at 1740 cm^{-1} in SF-PDA 200 Fe^{3+} without the disappearance of bands at 1453 cm^{-1} and 1110 cm^{-1} thus indicating that $FeCl_3$ curing affords the contemporary presence of both dihydroxyindole and indole-5,6-quinone which are responsible for adhesion and cohesion, respectively probably due to its chemical characteristic of an acidic oxidant.

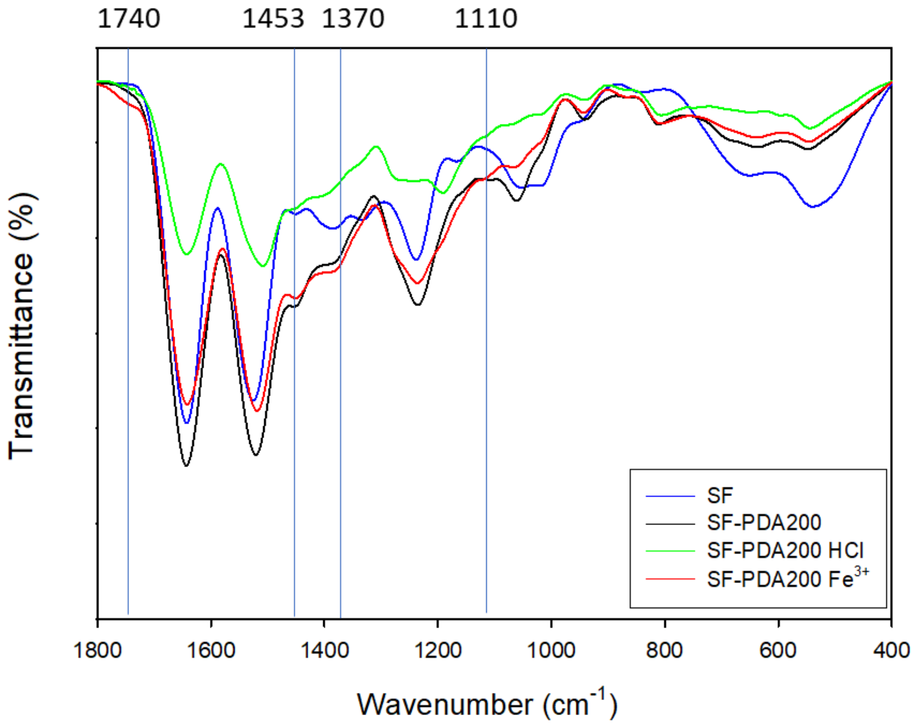

Figure S4. (FTIR-ATR spectra of SF (blue line), SF-PDA 200 (black line), SF-PDA 200 HCl cured (green line), SF-PDA 200 $FeCl_3$ cured (red line). Spectra were superimposed to show differences induced by curing.)

6. Experimental

6.1 UV-Vis Spectroscopy

A BioTek Synergy HT plate reader (Winooski, VT) was used to acquire UV-Vis spectra of Dopamine and silk-dopamine before and after polymerization.

6.2 Viscosity

Rheology measure was performed with a Brookfield DV-II+ Pro viscometer using a CPE-40 spindle at 50 rpm (20 °C).
500 µ*L* of SF solution with a concentration of 7 mg/m*L*, and SF solution with DA at 2, 20 and 200 mM were measured three times with a 24h intervals to show the difference in viscosity after dopamine polymerization. To show the increase of viscosity after the curing process, after 2 day of DA polymerization, to the solutions were added 14 µ*L* of either HCl 0.5 M or $FeCl_3$ 270 mM to maintain the same mass ratio between the curing agent and SF-PDA used in lap shear tests.
Data are reported as average ± SD of at least three measurements for each sample.

6.3 ATR-FTIR characterization

SF, SF-PDA 2, 20 and 200 mM and SF-PDA 200 mM after curing with HCl and $FeCl_3$ as for lap shear tests were characterized by Fourier Transformed Infrared Spectroscopy (FTIR) recorded by Bruker ALPHA II FTIR spectrometer, with an attenuated total reflectance unit (i.e., Platinum-ATR unit)

For each measurement, 128 scans were coadded with a resolution of 1 cm^{-1}, and the wavenumber ranged from 400 to 4000 cm^{-1}.

6.4 Lap shear tests

Lap shear tests were performed on 25x100x3.0 mm strips of Polished Multipurpose 304 Stainless Steel, Marine-Grade 5083 Aluminum and 8x50x2 mm of Marine-Grade Plywood Sheet.

For metal adherents 14 μL were casted on an area of 25x7 mm, while 10 μL were casted on an area of 15x8mm on wood specimens.

Adhesion tests were performed on an Instron 3366 testing frame equipped with a 1000 N load cell (Norwood, MA, USA) following a modified version of the ASTM method. Adherents mounted in the tension grips were subjected to single lap shear testing, repeating each measure at least five times. 1000 N load cell equipped Instron was programmed to move the grips apart at 0.5 mm/s rate. Steel specimens were blocked to the grips using a screw. Tests were stopped when adhesive bond ruptures and the maximum shear strength peak was divided by the bound area to give adhesion.

References

[1] D. Tuna, A. Udvarhelyi, A. L. Sobolewski, W. Domcke, T. Domratcheva, *J. Phys. Chem. B* **2016**, 120(14), 3493.
[2] M. Bisaglia, S. Mammi and L. Bubacco, *J. Biol. Chem.* **2007**, 282, 15597.
[3] S. Chen, S. Liu, L. Zhang, Q. Han, H. Liu, J. Shen, Y. Yang, *Chem. Eng. J.* **2020**, *399*, 125795.
[4] H. Pang, S. Zhao, L. Mo, Z. Wang, W. Zhang, A. Huang, J. Li, *J. Appl. Polym. Sci.* **2020**, (23), 48785.
[5] C. Chen, T. Yao, S. Tu, W. Xu, Y. Han and P. Zhou, *Phys. Chem. Chem. Phys.* **2016**, 18, 16353.
[6] S. A. Centeno, J. Shamir, *J. Mol. Struct.* **2008**, 873(1-3), 149.
[7] C. Zhijiang, H. Chengwei, *J. Power Sources* **2011**, 196, 10731.
[8] A. Banerjee, S. Supakar, R. Banerjee, *PLoS One* **2014**, 9, 1.
[9] T. An, N. Lee, H. J.Cho, S. Kim, D. S. Shin, S. M. Lee, *RSC adv.* **2017**, 7(49), 30582.
